# Pre-hospital care delays in trauma-related mass casualty incidents: a comparative international survey of high-income and low- and middle-income country perspectives

**DOI:** 10.1186/s13049-026-01664-8

**Published:** 2026-07-24

**Authors:** Fayez Alruqi, Malik A. Althobiani, Elaine Cole, Karim Brohi

**Affiliations:** 1 Emergency Medical Services Program, Department of Nursing, College of Nursing and Health Sciences, Jazan University, Jazan, Saudi Arabia; 2 Centre for Trauma Sciences, Blizard Institute, Queen Mary University of London, 4 Newark St, London, E1 2AT UK; 3 Department of Respiratory Therapy, Faculty of Medical Rehabilitation Sciences, King Abdulaziz University, Jeddah, Saudi Arabia

**Keywords:** Mass casualty incident, Pre-hospital, Pre-hospital time, Delay factors, Triage, Emergency medical services

## Abstract

**Background:**

Mass casualty incidents (MCIs) place substantial demands on pre-hospital systems. Extended pre-hospital times are associated with adverse patient outcomes. The factors perceived as contributing to these delays are poorly characterized across different resource settings. This study aimed to identify and compare pre-hospital care providers’ perceptions of factors associated with pre-hospital delays during trauma-related mass casualty incidents across high-income and low- and middle-income countries.

**Methods:**

A cross-sectional online survey was conducted between May and August 2024. Pre-hospital care providers with experience of at least one trauma-related mass casualty incident were recruited globally via professional associations, alumni networks, and social media. The survey comprised expert-reviewed Likert-scale items and open-ended questions. Participants were stratified by national income level using World Bank classifications. Quantitative data were analyzed using the Mann–Whitney U test and chi-squared or Fisher’s exact tests. Open-text responses were analyzed using thematic analysis following the framework of Braun and Clarke.

**Results:**

Of 225 eligible responses from 54 countries, 158 (70.2%) were from high-income countries and 67 (29.8%) from low- and middle-income countries. Poor communication and geographical challenges were the most frequently cited delay factors, with agreement rates of 84% and 80%, respectively. Agreement that equipment inadequacy contributed to delays was reported by 75% of respondents from low- and middle-income countries compared with 46% of respondents from high-income countries. Thematic analysis identified eight qualitative delay themes: communication and coordination issues, resource limitations, infrastructure and scene access challenges, hospital capacity and preparedness, administrative and policy issues, training and preparedness gaps, security and safety barriers, and responder emotional burden and stress. Administrative and policy barriers were reported exclusively by participants from high-income countries. Responder emotional burden and stress emerged solely from qualitative responses and lacked corresponding quantitative items. Regular training and prior mass casualty experience were widely endorsed as optimizing factors, with agreement rates of 95% and 90%, respectively.

**Conclusions:**

Clinician-perceived delay factors showed similar patterns across settings, particularly communication failures, while differing in emphasis by resource context. Communication improvement, integrated into routine MCI training and operational planning, may represent a modifiable area for reducing perceived pre-hospital delays.

**Supplementary information:**

The online version contains supplementary material available at https://doi.org/10.1186/s13049-026-01664-8.

## Introduction

A mass casualty incident (MCI) is an overwhelming event that leads to a substantial number of patients, exceeding the available resources and capabilities of local healthcare systems to respond effectively [1]. The analysis of historical records indicates an escalation in both the number and magnitude of MCIs globally over recent years [2]. For example, the United Nations Office for Disaster Risk Reduction reported 7,348 major disasters between 2000 and 2019, compared with 4,212 in the preceding two decades. Numerous countries across different income settings have experienced major MCIs in the last decade [3, 4]. These include terrorist attacks in high-income urban systems, such as the Manchester Arena bombing, and large-scale explosions in lower-resource settings, such as the Beirut port explosion [3, 4]. This has sparked a growing demand for information and accountability [5]. These concerns highlight the deficiencies in pre-hospital care during MCIs, particularly in terms of rapid critical care delivery at the scene.

Early pre-hospital response is vital to improving trauma patient outcomes [6]. To reduce the interval between injury and receipt of definitive care, modern trauma systems have introduced reforms such as regional trauma networks, pre-hospital triage protocols, and bypass pathways to major trauma centers [7, 8]. However, delivering early interventions during MCIs can be difficult. These situations often involve large numbers of casualties, limited resources, and operational pressure at the scene. Moreover, in an MCI, the priority shifts from achieving the best possible outcome for individual patients to achieving the best possible outcomes for the greatest number of patients [9]. In a review of contemporary MCIs, on-scene preventable deaths have been linked to delayed care [10]. MCI scene delays can be due to conventional challenges such as resource scarcity or long distances to definitive care. However, they may also result from unidentified factors present even in well-resourced urban settings.

To date, no studies have specifically explored pre-hospital care providers (PHCPs’) experiences of factors associated with MCI delay responses. Due to the unpredictable nature of MCIs, it is not feasible to conduct prospective studies to identify these factors. Consequently, much of the published literature relies on anecdotal evidence, often focusing on single incidents [4]. This approach does not provide a comprehensive view of global factors associated with delays as it is limited by the availability and scope of the gathered evidence. Therefore, this study aimed to identify and characterize clinician-perceived factors contributing to delays in pre-hospital care during MCIs, and to compare these perceptions across high-income and low- and middle-income country settings. A secondary aim was to explore PHCPs’ priorities for optimizing pre-hospital times (PHTs) and to determine whether these priorities differed by resource context.

## Methods

### Study design and setting

This study utilized a cross-sectional survey design to explore clinician-perceived factors contributing to delays in pre-hospital care during MCIs. The data were collected through an online questionnaire distributed globally to PHCPs between May and August 2024. This study was reported in accordance with the Consensus-Based Checklist for Reporting of Survey Studies (CROSS) (Additional file 1).

### Participants and eligibility criteria

Eligible participants were PHCPs from any professional background who had been involved in at least one trauma-related MCI within the preceding ten years. Eligibility was assessed through skip-logic questions at the start of the survey. Respondents were required to confirm both their PHCP role and previous MCI experience before proceeding to the survey. Responses were excluded if respondents did not confirm eligibility, did not report previous MCI experience, or met the eligibility criteria but did not complete the survey.

### Recruitment strategy

A convenience sampling strategy was used. PHCPs were invited through established pre-hospital care institutions and professional networks, including the National Ambulance Research Steering Group in the United Kingdom, the Australasian College of Paramedicine in Australia, and FIRST60: Pre-hospital, Trauma and Resuscitation Sciences in Canada. The survey link was also shared through MSc and PhD alumni from the Center for Trauma Sciences, Queen Mary University of London. Additional recruitment was conducted through ResearchGate and social media platforms, including X, Facebook, WhatsApp, and LinkedIn, to broaden international participation and reach PHCPs from different resource settings.

### Data collection tool

Data were collected using an English-language web-based survey hosted on SurveyMonkey (Portland, Oregon, USA). The survey comprised four sections. The first section was a skip-logic eligibility screen, through which only respondents who confirmed both their PHCP role and prior involvement in a trauma-related MCI within the preceding ten years were permitted to proceed. The second section collected demographic and MCI experience data, including country, education level, profession, years of experience, and MCI history. The third section presented two sets of Likert-scale items: twelve statements relating to factors perceived to extend PHTs, and ten statements relating to factors perceived to optimize PHTs. Each statement was a full declarative sentence describing a specific situation or condition, which participants rated on a five-point scale from “Strongly Disagree” to “Strongly Agree”, with an additional “Not Applicable” option for situations they had not encountered in their MCI experience. Each Likert set was followed by an open-text question inviting participants to describe any additional experiences not captured by the fixed items. The fourth section focused on triage system use and life-saving interventions during MCI response.

### Data collection and validation

The survey data were automatically collected via the hosting platform and subsequently transferred to an Excel file (Microsoft, Redmond, WA, USA) for analysis. To minimize potential errors, the exported data were checked and validated by the primary researcher. This process involved identifying duplicates, reviewing eligibility, checking completion status, and confirming whether the reported MCI experience met the study criteria.

### Ethical considerations

This study received ethical approval from the Devolved School Research Ethics Committee (DSREC) at QMUL, [reference: DSREC_01_v2_F_Alruqi]. In compliance with the Declaration of Helsinki, PHCPs provided their informed consent to participate in this study, with the consent form on the survey’s cover page outlining the voluntary nature of participation and ensuring anonymity. Participants did not receive any monetary or other incentives for their participation and had the opportunity to review their answers before finalizing their submission.

### Data analysis

For the quantitative data, the statistical data analysis was performed using RStudio 2023.9.1.494 (RStudio: Integrated Development Environment for R. Posit Software, PBC, Boston, MA, USA). The Shapiro–Wilk test was conducted to assess the normality of continuous data, revealing that most of the data were not normally distributed. Therefore, demographic information was summarized using medians with interquartile ranges or frequencies and percentages. To allow for meaningful interpretation of the data from differing settings, participants were categorized into two groups based on the national income level according to the World Bank classification: high-income countries (HICs) and Low- and middle-income countries (LMICs) [11]. Statistical comparisons for continuous data were performed using the Mann–Whitney U test. For categorical data, the Chi-squared test or Fisher’s exact test was used based on sample size. Given the cross-sectional and exploratory nature of this study, statistical comparisons between HIC and LMIC groups were treated as hypothesis-generating rather than confirmatory. A *p*-value of <0.05 was used as a threshold for flagging potentially meaningful differences between groups. The NA option was included for Likert-scale items to avoid forcing responses from participants who had not encountered the specific situation in their MCI experience. Accordingly, NA responses were excluded from the denominator when calculating agreement rates, as these participants could not meaningfully rate a situation they had not experienced. Agreement was defined as the combined proportion of “Agree” and “Strongly Agree” responses among substantive respondents. The effective denominators varied slightly across items, ranging from 217 to 224 out of 225 eligible participants, reflecting the small number of NA responses per item (range: 1–8).

The qualitative data from free text responses were analyzed using NVivo software (Version 14) (Lumivero, Denver, CO, USA). Thematic analysis was conducted following the six-phase framework described by Braun and Clarke, comprising: familiarization with the data, generation of initial codes, searching for themes, reviewing themes, defining and naming themes, and producing the final analysis [12]. The analysis was conducted using an inductive, semantic approach, focusing on explicit meanings within the data. Only responses containing substantive content relevant to the question were included as codable responses; brief or non-informative entries were excluded. Initial coding and theme development were conducted by the primary author (FA). Preliminary codes were generated inductively from repeated concepts within the open-text responses and were refined iteratively during analysis. A coding framework was developed during this process and updated as coding progressed. Related codes were grouped into subthemes and broader themes. No minimum numerical threshold was applied for theme generation, as themes were developed based on repeated and clinically relevant concepts rather than frequency alone. All subthemes and their descriptions were derived inductively from participant free-text responses and were not predefined in the survey instrument. An independent review of the coding framework and emergent themes was subsequently performed by a second author (MA) to ensure consistency and analytical rigor, with discrepancies resolved through discussion until consensus was reached. The percentage of each theme was calculated based on the total number of participants who provided codable responses to the open-text question. For group comparisons, percentages were calculated based on the total number of participants within each group who responded to the open-text question with codable answers.

## Results

**Fig. 1:**
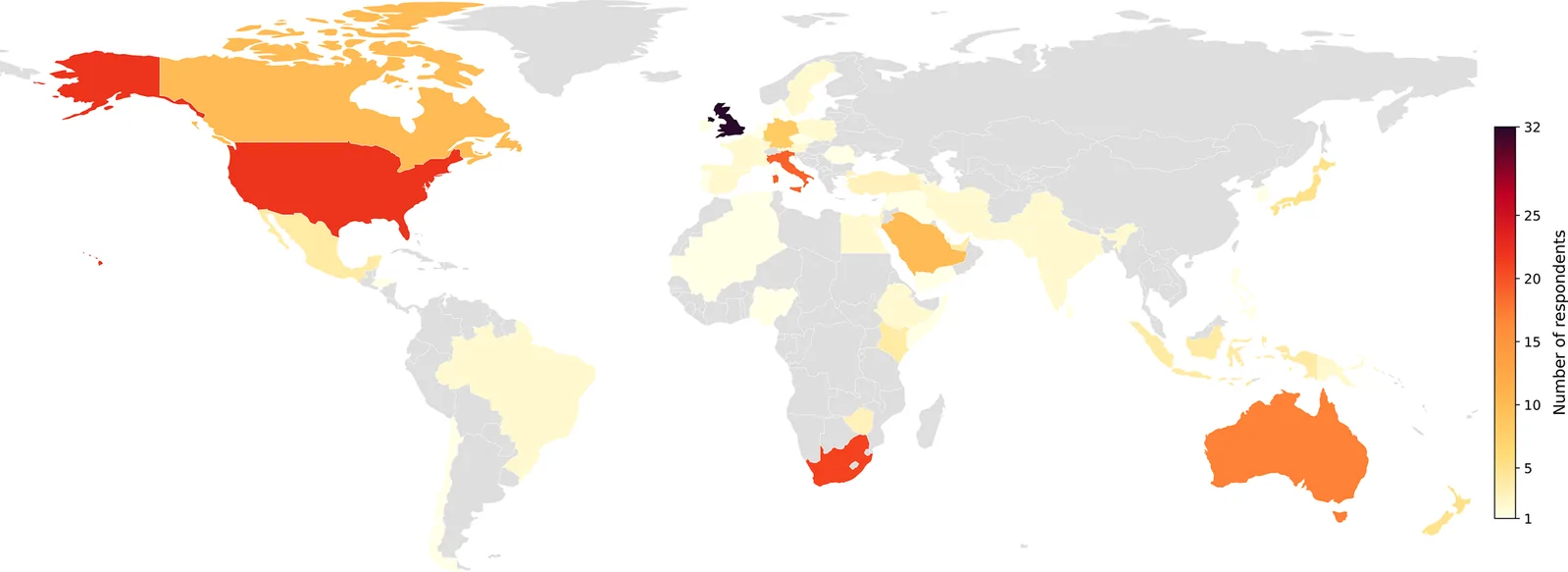
The global distribution of survey respondents across different countries. The map shows the number of survey respondents per country (*N* = 225). A color gradient from light yellow to deep purple indicates increasing respondent counts (range: 1–32). White regions represent countries with no respondents

Of the 444 respondents who opened the survey, 225 (50.7%) eligible responses were included in the final analysis. A total of 214 (48.2%) responses were excluded because respondents did not confirm eligibility, did not report previous MCI experience, or met the eligibility criteria but did not complete the survey. A further 5 (1.1%) responses were excluded during data validation because the reported MCI experience was not trauma-related or was based on an exercise rather than an actual incident. Included responses came from 54 countries, with the largest proportion from Europe (33.8%), followed by Asia (20.9%), North America and Africa (each 16.4%), Oceania (10.7%), and the least from South America (1.8%) (Fig. 1). The majority of participants were paramedics (62.2%), followed by physicians (16.4%), emergency medical technicians (EMTs) (7.6%), nurses (5.3%), and other roles (8.4%), which included firefighters and police officers, EMS directors, commanders, disaster managers, hazmat specialists, academic personnel, volunteers, and dual-qualified professionals (Table 1). Differences were observed between the two groups in terms of roles and education levels, with HIC respondents being more likely to have higher educational qualifications (*p* = 0.046). Additionally, participants had varying years of experience, with HIC respondents reporting a median of 15 years, compared to 8 years in LMICs (*p* < 0.001).

**Table 1:** Demographic and MCI experience of survey participants

Category	Subcategory	HIC (158) N (%), Median IQR	LMIC (67) N (%), Median IQR	All (225) N (%), Median IQR	*p* value*
**Roles**	Emergency Medical Technician	6 (3.80%)	11 (16.4%)	17 (7.56%)	**0.018**
	Paramedic	99 (62.7%)	41 (61.2%)	140 (62.22%)	
	Physician	29 (18.4%)	8 (11.9%)	37 (16.44%)	
	Nurse	9 (5.70%)	3 (4.48%)	12 (5.33%)	
	Other**	15 (9.5%)	4 (6.0%)	19 (8.4%)	
**Education**	Diploma	22 (13.9%)	18 (26.9%)	40 (17.78%)	**0.046**
	Undergraduate degree	43 (27.2%)	23 (34.3%)	66 (29.33%)	
	Master’s degree	56 (35.4%)	15 (22.4%)	71 (31.56%)	
	Doctoral degree	29 (18.4%)	7 (10.4%)	36 (16.00%)	
	Other	8 (5.06%)	4 (5.97%)	12 (5.33%)	
**Years of Experience**	-	15 (IQR 10–25)	8 (IQR 4–15)	13 (IQR 8–25)	**<0.001**
Area of Service***	Urban area	105 (66.5%)	47 (70.2%)	152 (67.56%)	0.700
	Suburban area	88 (55.7%)	31 (46.3%)	119 (52.89%)	0.250
	Rural area	71 (44.9%)	25 (37.3%)	96 (42.67%)	0.363
	Isolated or natural area	26 (16.5%)	14 (20.9%)	40 (17.78%)	0.545
**Number of MCI Experience**		5 (IQR 3–10)	10 (IQR 5–20)	5 (IQR 3–14)	**<0.001**
**Last MCI Response**	Less than 6 months ago	39 (24.7%)	27 (40.3%)	66 (29.3%)	**<0.001**
	Less than 1 year ago	31 (19.6%)	23 (34.3%)	54 (24.0%)	
	Less than 2 years ago	22 (13.9%)	7 (10.4%)	29 (12.9%)	
	Less than 3 years ago	13 (8.23%)	1 (1.49%)	14 (6.22%)	
	More than 3 years ago	52 (32.9%)	8 (11.9%)	60 (26.7%)	
	I don’t know	1 (0.63%)	1 (1.49%)	2 (0.89%)	

Abbreviations: HIC; High-Income Country, LMIC; Low- or Middle-Income Country

*p-values are based on Fisher’s Exact Test or Chi-Square Test (for categorical data) and Mann-Whitney U Test (for continuous data)

**Other roles included firefighters and police officers (*n* = 8) and specialist or administrative roles, including EMS directors, commanders, disaster managers, hazmat specialists, academic personnel, volunteers, and dual-qualified professionals (*n* = 11)

***For questions where more than one answer could be chosen, separate *p*-values were calculated for each category using Fisher’s Exact Test or Chi-Square Test

Given the study’s comparative aims, all subsequent analyses are presented both overall and stratified by income group, to highlight the contrasting experiences and priorities of PHCPs in different resource contexts.

Participants from LMICs reported a significantly higher median number of MCI experiences (10: IQR 5–20) compared to those from HICs (5: IQR 3–10, *p* < 0.001) (Table 1). Given the higher number of MCI experiences reported by LMIC respondents, it follows that a greater proportion also reported more recent MCI responses compared to those from HICs (40.3% vs 24.7%, *p* < 0.001).

In terms of the types of MCIs experienced, transport accidents were the most commonly reported by both settings (83.11%), with no significant differences across the groups. However, structural collapses, mass shootings, and stampedes were significantly more common among LMIC respondents (*p* = 0.028, *p* = 0.025, and *p* = 0.030, respectively) (Additional file 3).

When examining the number of casualties, the most common size of incident involved 11–30 victims, reported by 25.8% of participants (Additional file 3). In addition, significant differences were observed between HIC and LMIC groups in incidents involving 11–30 victims (*p* = 0.036), 31–50 victims (*p* = 0.039), and 51–200 victims (*p* = 0.0047) (Additional file 3). Participants reported on-scene time as the longest time interval in pre-hospital MCI care, with a significantly higher proportion in HICs (72.2%) compared to LMICs (43.3%) (*p* < 0.001) (Additional file 4). In contrast, response time was more frequently reported as the longest interval in LMICs (43.3%) than in HICs (16.5%), while transport time showed minimal variation.

### Clinician-perceived factors extending pre-hospital time in trauma-related mass casualty incidents

#### Overall Likert scale results

**Fig. 2:**
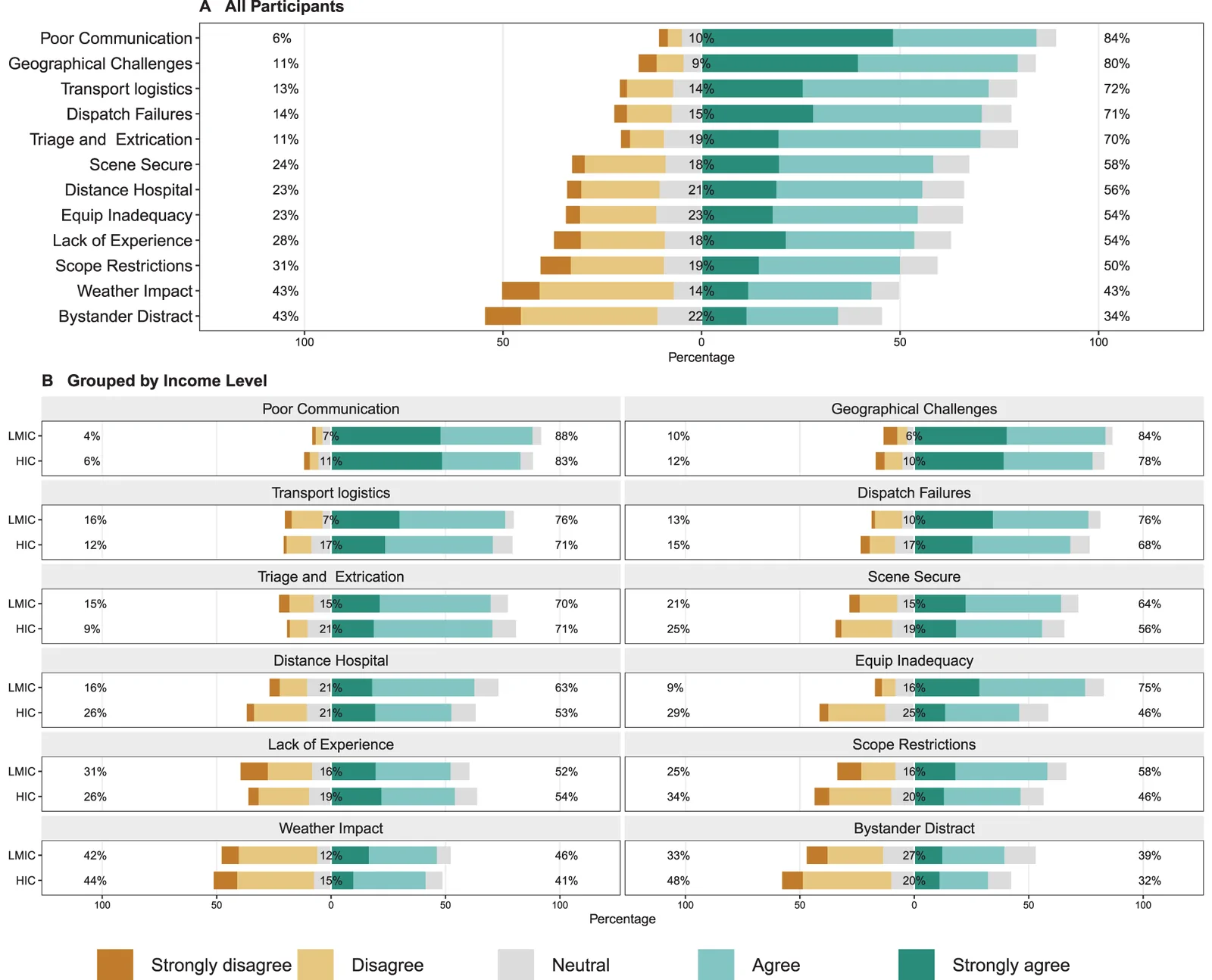
Respondents’ perceptions of delay factors in MCIs. Panel **A** presents the overall agreement levels across all participants, while Panel **B** displays the same delay factors grouped by country income level (high-income countries vs. low- and middle-income countries). Percentages are calculated based on respondents who provided valid (non-NA) responses

Respondents reported that poor communication and geographical challenges were perceived as the primary factors extending PHTs in MCIs, with high levels of agreement at 84 and 80%, respectively (Fig. 2.A). Other factors which achieved levels of agreement of 70% or above included transport logistics, dispatch failures and triage, and extrication. The impact of weather and bystander distractions had the lowest levels of agreement, 43 and 34%, respectively.

The Likert-scale analysis by income group revealed that, for most factors, there were no significant differences between HIC and LMIC respondents in their agreement or disagreement on the causes of delays (Fig. 2.B). However, equipment inadequacy showed the largest disparity, with 75% of LMIC respondents agreeing compared to 46% of HIC respondents, while disagreement was 20% higher among HIC respondents (29% vs 9%). Similarly, the distraction caused by bystanders had 15% more disagreement among HIC participants compared to LMIC respondents (48% vs 33%). Lastly, scope restriction saw more agreement in LMICs than in HICs, with a 12% difference (58% vs 46%).

#### Qualitative insights

**Fig. 3:**
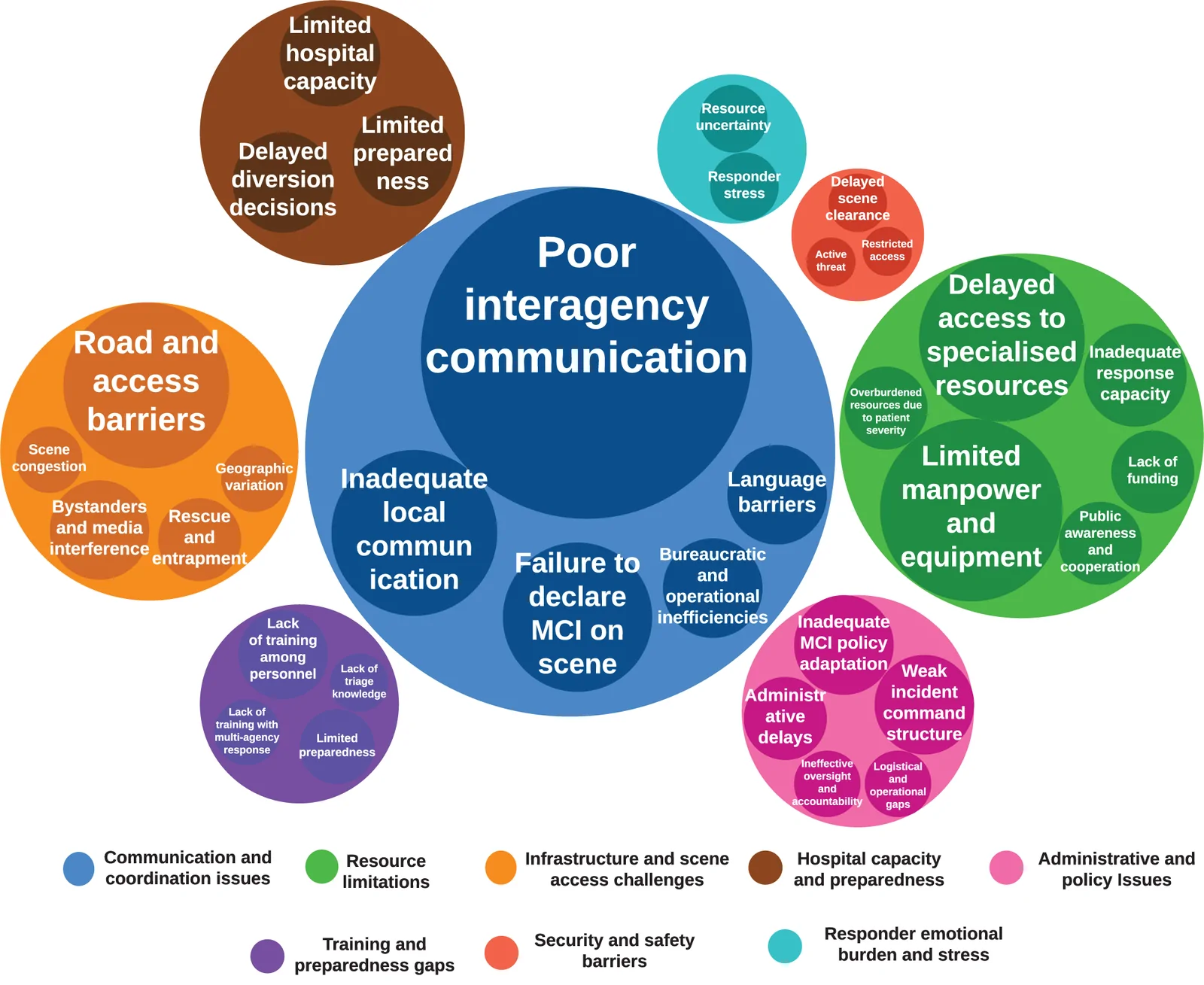
Clustered bubble chart of reported delay factors in MCI pre-hospital care. The outer circles represent main themes, while the inner bubbles represent sub-themes, with bubble size reflecting how frequently each theme and subtheme was mentioned by participants in the open-text responses

Eight themes emerged from the open-text questions regarding delays in PHTs (Fig. 3), and their correspondence with quantitative Likert findings is summarized in Table 2.

**Table 2:** Qualitative themes and corresponding quantitative Likert findings for clinician-perceived pre-hospital delay factors

Qualitative theme	Qualitative frequency	Corresponding quantitative factor	Overall agreement	LMIC agreement	HIC agreement	Notable difference
Communication and coordination issues	30.3%	Poor communication	84%	88%	83%	Minimal
Infrastructure and scene access challenges	18.4%	Geographical challenges	80%	84%	78%	Minimal
Infrastructure and scene access challenges	18.4%	Transport logistics	72%	76%	71%	Minimal
Communication and coordination issues	30.3%	Dispatch failures	71%	76%	68%	Moderate
Training and preparedness gaps	10.0%	Triage and extrication	70%	70%	71%	Minimal
Security and safety barriers	7.9%	Scene security	58%	64%	56%	Moderate
Resource limitations	22.4%	Distance to hospital	56%	63%	53%	Moderate
Resource limitations	22.4%	Equipment inadequacy	54%	75%	46%	Large
Training and preparedness gaps	10.0%	Lack of experience	54%	52%	54%	Minimal
Resource limitations	22.4%	Scope restrictions	50%	58%	46%	Moderate
Infrastructure and scene access challenges	18.4%	Weather impact	43%	46%	41%	Minimal
Administrative and policy issues	HIC only	No quantitative equivalent	—	—	—	HIC exclusive
Hospital capacity and preparedness	Qualitative only	No quantitative equivalent	—	—	—	Qualitative only
Responder emotional burden and stress	6.6%	No quantitative equivalent	—	—	—	Qualitative only

Abbreviations: HIC, high-income countries; LMIC, low- and middle-income countries; MCI, mass casualty incident. Agreement defined as combined “Agree” and “Strongly Agree” responses. —, no corresponding quantitative item

##### Communication and coordination issues

In the open-text responses, communication and coordination issues were reported by 30.3% of participants who provided codable responses. The main subthemes were poor interagency coordination, poor local communication, delayed declaration of an MCI, operational inefficiencies, and language barriers. Poor interagency coordination was the most frequently reported subtheme, particularly in relation to communication between ambulance services, hospitals, physicians, fire services, and command teams. One participant described this as *“poor communication with EMS, Hospital, Physician”.* Delayed declaration of an MCI was also reported as a source of delay, particularly when recognition of the incident was delayed by the first responding team or dispatch center. Language barriers were less frequently reported, but were included when participants described difficulties communicating with patients, bystanders, or responders during the incident.

##### Resource limitations

Resource limitations were the second most frequently reported factor, mentioned by 22.4% of participants, with a higher proportion reported by PHCPs in LMICs. Six subthemes were identified, with shortages of personnel and equipment being the most commonly reported. One participant stated, *“Limited manpower, especially in the first 30 minutes after the incident, is a contributing factor to delays”.* Delayed access to specialized resources was also reported, including delays in accessing helicopter emergency medical services and additional resources from distant locations. Inadequate response capacity was another subtheme, reflecting concerns that basic and advanced life support units were not designed to manage large numbers of injured patients. Other less frequently reported subthemes included lack of funding, limited public awareness and cooperation, and resource strain related to patient severity.

##### Infrastructure and scene access challenges

Infrastructure and scene access challenges were reported by 18.4% of PHCPs. The main subthemes were road and access barriers, scene congestion and blocked egress, public and media interference, search and rescue delays, and geographic variation in system performance. Road and access barriers included poor road conditions, rural gravel roads, potholes, road closures, and disruption to the road network caused by the incident. Scene congestion and blocked egress referred to situations where emergency vehicles or other responders obstructed routes for patient evacuation. Public and media interference included bystanders, media, or political figures disrupting access to the scene. Search and rescue delays were reported when patients were difficult to locate or were trapped, such as during rail incidents. Geographic variation in system performance referred to differences in response, scene, transport, and hospital acceptance times across different locations.

##### Hospital capacity and preparedness

Hospital capacity and preparedness was reported in 12 open text responses. These responses described limited hospital capacity, delayed diversion decisions, and a lack of preparedness to receive multiple casualties. One participant noted, *“Hospital reached max capacity and did not inform for diversion to next nearest hospital”.* Another participant described the challenge as hospitals being *“not prepared or properly trained to receive many victims. Noticeably, once you pass over your fourth victim, the system internally tends to crumble”.*

##### Administrative and policy issues

Ten of the PHCPs reported that current policies and administrative practices contributed to delays in PHT, with all such comments originating from participants in HICs. Five subthemes were articulated from these issues, with poor MCI policy adaptation and a weak incident command structure being the most frequently mentioned. For inadequate MCI policy, most comments focused on the lack of protocols, strategies, or operational plans. In terms of a weak incident command structure, participants highlighted how fragile their systems were, leading to expected delays in PHT.

Administrative delays were highlighted as a subtheme, with one participant noting that *“red tape and policy issues are usually the biggest contributing factors to delays at mass casualty incidents”*. Two additional subthemes were also identified: gaps in logistical and operational management, and the absence of effective oversight and accountability, with the latter contributing to the recurrence of these issues.

##### Training and preparedness gaps

Training and preparedness gaps were reported by one in ten participants who answered the open-text question. The main subthemes were lack of training among personnel, limited preparedness, lack of triage knowledge, and limited multi-agency training. Lack of training among personnel accounted for half of the responses within this theme. Limited preparedness was retained as a separate subtheme when participants described broader readiness issues rather than training alone.

##### Security and safety barriers

Security and safety barriers were reported by 7.9% of participants in the open-text responses. The main subthemes were delayed scene clearance, unsafe or active threat environments, and restricted access to casualties. Delayed scene clearance referred to situations where EMS access was delayed until law enforcement confirmed that the scene was safe. One participant noted, *“Law enforcement was slow to clear EMS to access patients during shooting events”.* Unsafe or active threat environments included ongoing threats to responders and patients, including violence or continued attacks on ambulance teams.

##### Responder emotional burden and stress

Responder emotional burden and stress was the least frequently reported theme, reported by 6.6% of participants. This theme was identified only in the qualitative data and had no corresponding quantitative item. Two subthemes were identified: situational awareness and resource uncertainty, and responder emotional burden. Situational awareness and resource uncertainty referred to a limited real-time understanding of the size of the incident, the number of casualties, and the resources required. Responder emotional burden referred to anxiety, stress, and difficulty managing patients during high-pressure MCI situations. One participant stated, *“When we encounter a patient who requires intensive care and our colleague becomes anxious and overwhelmed by seeing such a patient, it becomes even more challenging to manage the patient”.*

### Clinician-perceived factors optimizing pre-hospital time in trauma-related mass casualty incidents

#### Overall Likert scale results

**Fig. 4:**
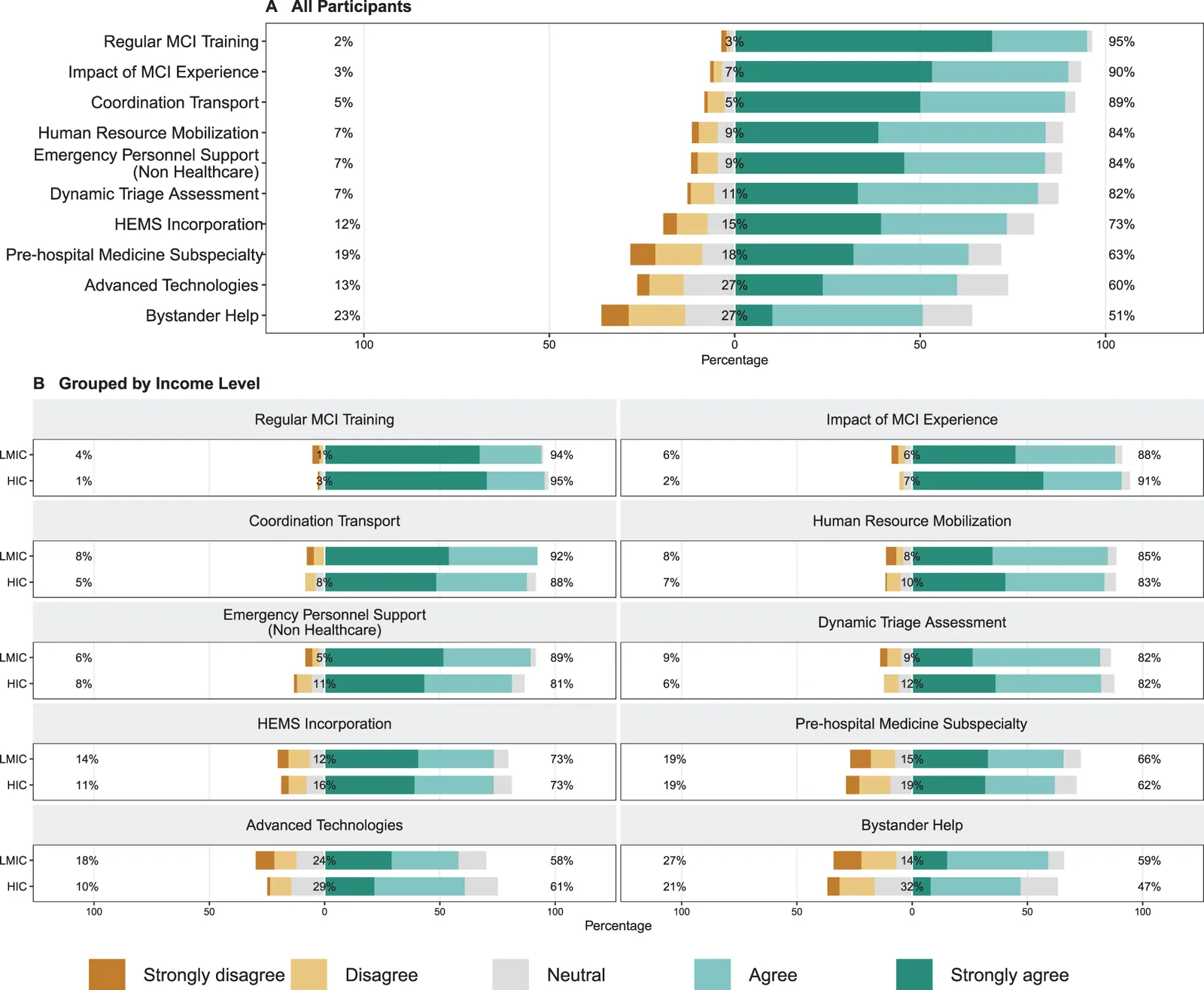
Respondents’ perceptions of factors that help reduce pre-hospital time during MCIs. Panel **A** presents the overall agreement levels across all participants, while Panel **B** displays the same factors grouped by country income level (high-income countries vs. low- and middle-income countries). Percentages are calculated based on respondents who provided valid (non-NA) responses

PHCPs reported high agreement on factors that help reduce PHTs during MCIs, with regular training receiving the highest agreement at 95% (Fig. 4.A). This was followed by frequent response to MCIs (90%) and improved coordination of patient transport (89%). Conversely, bystander assistance at the scene (51%) and the use of advanced technology (60%) received the least agreement as factors improving PHTs during MCIs.

The Likert-scale analysis by income groups revealed close agreement and disagreement between HIC and LMIC respondents, with most factors showing no more than an 8% difference (Fig. 4.B). However, a clear difference was observed in the perception of bystander help during MCIs, with LMIC participants showing 12% more agreement compared to their HIC counterparts (59% vs 47%).

#### Qualitative insights

**Fig. 5:**
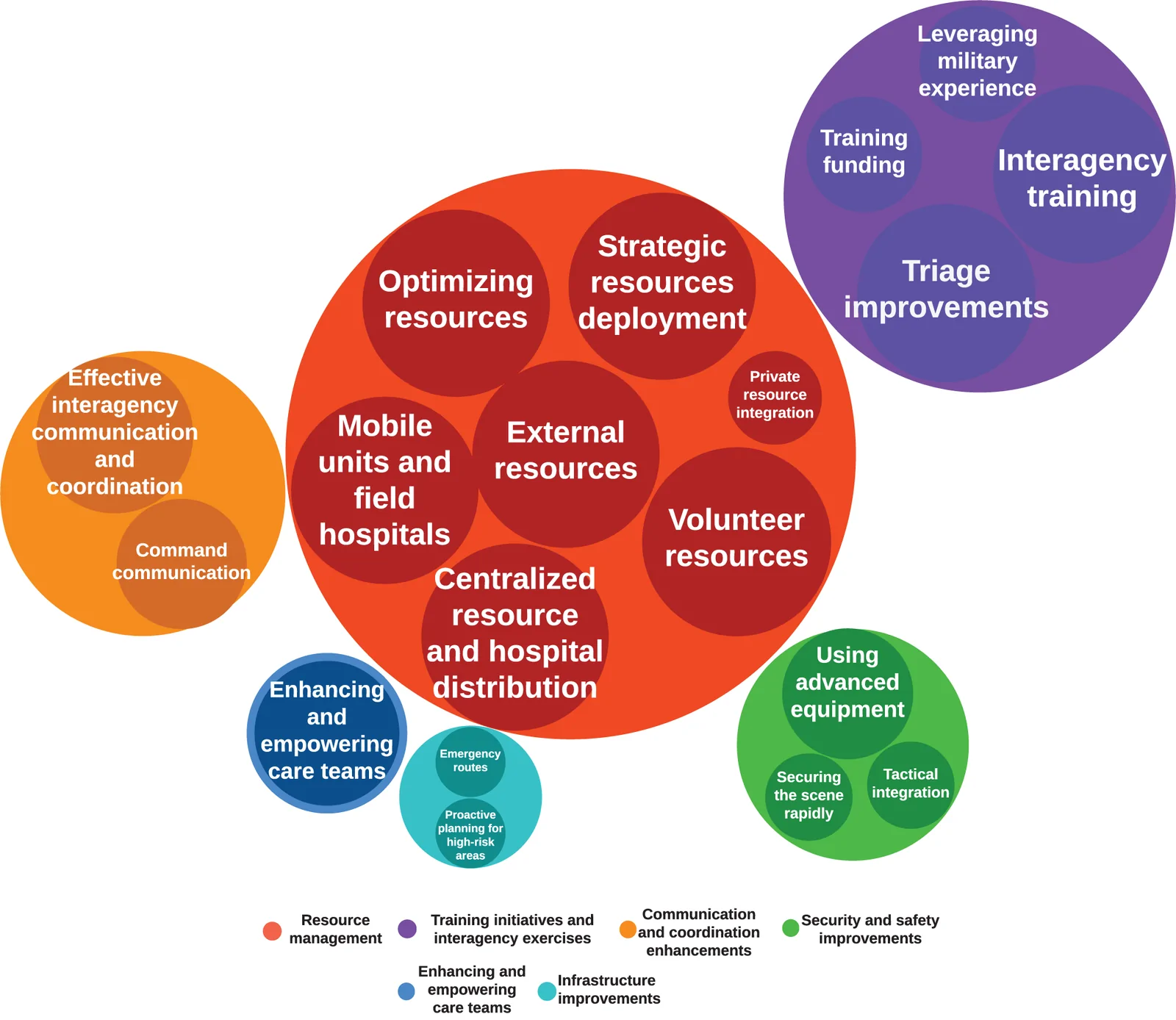
Clustered bubble chart of reported factors that may reduce pre-hospital delays in MCI care. The outer circles represent main themes, while the inner bubbles represent sub-themes, with bubble size reflecting how frequently each theme and subtheme was mentioned by participants in the open-text responses

Six main approaches were emphasized based on PHCPs’ experiences of the core aspects needed to accelerate response times in MCIs (Fig. 5).

##### Resource management

Resource management was the most frequently reported approach for reducing pre-hospital delays, reported by 13 participants (40.6% of codable responses). The main subthemes were optimizing existing resources, strategic resource deployment, centralized resource coordination, mobile units and field hospitals, use of external resources, and integration of private or volunteer resources. Optimizing existing resources included transporting more than one patient in a single ambulance when appropriate. Strategic resource deployment included positioning or dispatching available resources to reduce response time. Centralized resource coordination focused on distributing resources and patients across hospitals. Mobile units and field hospitals were described as ways to bring care closer to the scene, particularly during prolonged MCIs. External resources included off-duty medical providers, volunteers, and helicopter emergency medical services. Integration of private or volunteer resources referred to collaboration between public services and non-public response assets.

##### Training initiatives and interagency exercises

Training initiatives and interagency exercises were raised by a quarter (25%) of participants. Several core initiatives were recognized, with most emphasizing triage improvements. Different participants reported the need to enhance current triage systems to make them more precise, while others recommended simplified triage pathways to improve the rapid delivery of care. Another subtheme highlighted was the need for interagency training, which was highly emphasized. One participant remarked, *“Train, train, train! The more exposure you have and the stronger the interagency relationships you build, the greater your ability to perform during a mass casualty event”.* Another singularly reported solution was to enhance funding for training and learn from military experience.

##### Communication and coordination enhancements

Improvement in communication and coordination was reported by 18.8% of participants. The main subthemes were enhanced interagency communication and improved local command communication. This theme was emphasized only by participants from high-income countries, with no responses from LMICs. Enhanced interagency communication focused on communication between pre-hospital care services and hospital receivers. Improved local command communication focused on clear and precise commands from leadership.

##### Security and safety improvements

Security and safety improvements were reported by 12.5% of participants and were predominantly reported by participants from LMICs. The main subthemes were the use of advanced equipment, rapid scene security, and medical and police tactical integration. The use of advanced equipment in active threat or unsafe scenes was emphasized, with one participant noting that night vision goggles were helpful in improving response time. Another participant highlighted the benefits of using drones: *“Setting up a drone view on scene may help assess safety and patient deterioration in the triage and treatment area. The safety officer will have an advantage, overseeing like a bird’s-eye view, especially in open MCIs”.* Rapid scene security referred to responses describing faster scene clearance or earlier security coordination before PHCPs could access casualties. Medical and police tactical integration focused on PHCPs who had received tactical training to collaborate with police during scene security, enabling them to provide urgent treatment and prevent further delays.

##### Enhancing and empowering care teams

Enhancing and empowering care teams was reported by 3 participants (9.4% of codable responses), all from HICs. Participants described the need for improvements in personnel and decision-making processes. They highlighted the need to optimize medical teams’ decisions, particularly by empowering clinicians on scene to make decisions without waiting for control from those not present. This improvement also called for establishing dedicated medical leadership within police and fire and rescue services, as well as enhancing the care teams themselves.

##### Infrastructure improvements

Infrastructure improvements were reported by 6.3% of participants and were the least mentioned theme. The main subthemes were dedicated emergency routes and proactive planning for high-risk sites. Dedicated emergency routes were suggested to improve access for emergency vehicles entering or leaving the scene. Proactive planning for high-risk sites was also mentioned, including concerns about limited ambulance crews and equipment at locations such as airports.

### Pre-hospital triage and response

Pre-hospital triage systems were widely used, with participants reporting more than 18 different systems. Simple Triage and Rapid Treatment (START) was the most commonly reported system (*n* = 104, 46.43%), followed by the Standard Sieve (*n* = 31, 13.8%). Overall, 164 of 225 PHCPs (72.8%) reported that the triage system they used was effective. Reported strengths included simplicity, ease of use, prioritization of care, familiarity, standardization, and rapid application. Reported limitations included complexity, limited flexibility, reliance on clinical judgment, slow application, and limited adaptability to different incident types or scales (see Additional file 5).

Participant responses regarding life-saving interventions indicated that basic interventions were more likely to be implemented during triage, whereas advanced interventions were less commonly performed. Tourniquet application (*n* = 150, 66.66%) and direct pressure for bleeding (*n* = 134, 59.56%) were among the interventions more likely to be implemented, while surgical cricothyroidotomy (*n* = 97, 43.11%), chest tube insertion (*n* = 90, 40.00%), rapid sequence intubation (*n* = 76, 33.77%), and finger thoracostomy (*n* = 47, 20.89%) were among those less likely to be implemented (see Additional file 6).

## Discussion

This international survey found that PHCPs perceived both shared and context-specific factors associated with pre-hospital delays during trauma-related MCIs. Communication failures and geographical challenges were commonly reported across settings, while equipment inadequacy showed the clearest difference between income groups, with higher agreement among LMIC respondents. The qualitative findings also identified context-specific concerns, including administrative and policy issues reported by HIC respondents and resource limitations reported more frequently by LMIC respondents. These findings suggest that MCI preparedness may need to consider the specific barriers reported in each setting, including communication, geography, equipment availability, hospital distance, scope of practice, and administrative processes.

Communication challenges are well-documented within MCIs and can affect patient care [13, 14]. In the present study, communication and coordination issues were among the most frequently cited factors in both the quantitative and qualitative findings, particularly poor interagency coordination, poor local communication, and delayed MCI declaration. This aligns with reviews of recent MCIs, including terrorist incidents, where communication failures were repeatedly identified as a major challenge during pre-hospital response [13, 14]. Poor communication may also contribute to secondary delays through inappropriate resource deployment, unclear command structures, and mismanagement of triage priorities [13, 15, 16]. These findings suggest that communication planning should remain an important component of MCI preparedness, particularly through joint interagency exercises [13, 15, 16].

Geographical challenges were the second most frequently cited factor associated with extended PHT. This aligns with a previous systematic review conducted as part of this research, which identified geographic barriers as an important contributor to delayed pre-hospital care during trauma-related MCIs [4]. In the present study, these challenges were reflected in both the quantitative findings and the qualitative theme of infrastructure and scene access challenges, including road and access barriers, scene congestion, and search and rescue delays where victims were trapped or difficult to locate.

Resource limitations were more frequently reported by LMIC respondents, particularly equipment inadequacy, hospital distance, and restrictions in the scope of practice. This is consistent with reports from low-resource settings, where limited supplies, staffing constraints, and fragile infrastructure can hinder MCI response and extend pre-hospital care [17, 18]. The 2020 Beirut explosion illustrates how damaged hospitals, shortages of medical supplies, and overwhelmed facilities can increase reliance on longer patient transfers and external assistance [18]. These findings suggest that resource-focused preparedness, including equipment availability and coordinated mutual aid arrangements, may be particularly relevant in lower-resource settings [19].

Administrative and policy issues were reported only by HIC respondents in the qualitative findings, suggesting that organizational barriers may contribute to delays even in well-resourced systems. Previous reports from MCIs have described similar challenges, including unclear coordination, ambiguous role allocation, and difficulties managing off-duty or volunteer responders without clear command structures [20]. These findings suggest that resource availability alone may not ensure timely MCI response unless supported by clear governance, defined roles, and effective coordination systems.

Regular preparedness training and prior MCI experience were widely endorsed as factors that may help optimize PHT during MCIs, with agreement rates of 95 and 90%, respectively. This aligns with educational literature suggesting that recent and repeated training can improve clinicians’ perceived readiness [21]. Discussion-based, operation-based, and interagency exercises may therefore support MCI preparedness, particularly in HICs where direct MCI exposure may be less frequent. The greater agreement among LMIC respondents regarding bystander assistance (59% vs 47% in HICs) may also reflect the importance of informal or community support when professional resources are limited [22].

Security and safety improvements were reported more frequently by LMIC respondents as factors that may help optimize PHT. This may reflect the operational challenges of responding to MCIs in settings where scene safety, active threats, or delayed access to casualties can limit the ability of PHCPs to reach patients. Previous work in resource-limited MCI training also highlights the importance of adapting preparedness approaches to local risks and available resources [16]. These findings suggest that security planning, including coordination with law enforcement during MCI exercises, may be an important consideration in lower-resource settings.

Triage systems are intended to support rapid prioritization of casualties during MCIs, particularly when resources are limited, and scene decisions must be made quickly [23]. In this study, most PHCPs reported that their triage system was effective, although qualitative responses highlighted limitations related to complexity, flexibility, and reliance on clinical judgment. These concerns align with broader discussions that disaster triage tools should be simple, rapid, and adaptable to different incident contexts [24].

Future research could combine the clinician-perceived delay factors identified in this survey with quantitative data on pre-hospital time intervals, life-saving intervention timings, casualty flow, and resource availability to inform computer simulation models of MCI response. This approach may help test how modifiable factors, such as communication delays, resource allocation, and intervention timing, influence overall scene time and patient outcomes. Such modeling could build on existing life-saving intervention timing data and evaluate different preparedness strategies before real world implementation [25].

### Limitations

This study has several limitations. Confidentiality restrictions and social media recruitment may have limited the response rate. The use of convenience sampling and voluntary participation may have introduced selection bias, and wider recruitment through additional trauma, emergency medicine, and disaster medicine organizations may have improved representativeness. The sample included more respondents from HICs than LMICs, which may limit the generalizability of the findings to lower-resource settings. The study was not powered to detect differences between income groups, so comparisons between HIC and LMIC respondents should be interpreted as exploratory. Grouping low- and middle-income countries together may also have masked differences between low-income, lower-middle-income, and upper-middle-income settings. Differences between HIC and LMIC respondents may partly reflect participant characteristics, including years of experience, professional role, and prior MCI exposure, rather than income setting alone. The study relied on self-reported data, which may have introduced recall bias and subjective interpretation, particularly because MCIs are rare and infrequent events. Finally, the survey was conducted in English only, which may have affected participation from some non-English-speaking PHCPs.

## Conclusion

This study suggests that the factors associated with pre-hospital delays during MCIs are multifactorial, spanning communication, resource availability, infrastructure, training, and organizational coordination. Although the importance of each factor varied across settings, communication and regular training were commonly reported by participants in both high- and lower-resource settings and may represent useful starting points for improvement. Preparedness efforts may be strengthened by involving frontline clinicians in their design, so that the resulting strategies reflect the practical realities and constraints of pre-hospital systems in different settings.

## Electronic supplementary material

Below is the link to the electronic supplementary material.

Supplementary Material 1. Additional file 1: CROSS Checklist (.docx). Completed Consensus-Based Checklist for Reporting of Survey Studies checklist for the current study

Supplementary Material 2. Additional file 2: Questionnaire items (.pdf). Survey questionnaire items used for data collection, including Likert-scale statements and open-ended questions

Supplementary Material 3. Additional file 3: Comparison of MCI types and casualty counts (.docx). Distribution of mass casualty incident types and casualty sizes reported by participants, stratified by income group

Supplementary Material 4. Additional file 4: Longest pre-hospital time intervals (.docx). Participant-reported data on the longest pre-hospital time interval experienced during mass casualty incidents, stratified by income group

Supplementary Material 5. Additional file 5: Triage effectiveness (.docx). Participant-reported data on pre-hospital triage system use and perceived effectiveness during mass casualty incident responses

Supplementary Material 6. Additional file 6: Pre-hospital interventions (.docx). Participant-reported data on basic and advanced life-saving interventions performed during mass casualty incident responses

## Data Availability

No datasets were generated or analysed during the current study.

## Abbreviations

CROSS: Checklist for Reporting of Survey Studies
MCI: Mass Casualty Incident
PHCP: Pre-hospital Care Providers
PHT: Pre-hospital Time
HIC: High-Income Countries
LMIC: Low- and Middle-Income Countries
