# Supplementary material for: Pre-hospital care delays in trauma-related mass casualty incidents: a comparative international survey of high-income and low- and middle-income country perspectives
Source: Scand J Trauma Resusc Emerg Med. 2026 Jul 24;34:166. doi: 10.1186/s13049-026-01664-8 (PMC13692905; doi:10.1186/s13049-026-01664-8)
Supplement: Supplementary file 1 [file 13049_2026_1664_MOESM2_ESM.pdf]

Invitation to Participate:
If you are involved in pre-hospital care, your expertise is invaluable to us. We invite you to participate in a research study conducted by mass casualty experts Professor Karim Brohi, Professor Elaine Cole, and Mr. Fayez Alruqi at the Centre for Trauma Sciences, Queen Mary University of London.

The study focuses on identifying the factors that cause delays in pre-hospital care during mass casualty incidents, with the ultimate goal of improving response times and patient outcomes. For more detailed information about the study and its processes, please click here.

Queen Mary Ethics of Research Committee Ref: DSREC_01_v2_F_Alruqi

By clicking Next and submitting the survey, you are providing your informed consent.

* Have you worked as a pre-hospital care provider and responded to trauma-related mass casualty incidents?

The term Mass Casualty Incident (MCI) is defined by the World Health Organisation as a short-term event that overwhelms the local medical care systems with many patients that exhaust the available resources and capabilities.

- ◯ Yes
- ◯ No

Demographic section

* 1.1 Please specify your role in pre-hospital care:

- ◯ First responder (e.g., firefighter, police officer)
- ◯ Emergency Medical Technician (EMT)
- ◯ Paramedic
- ◯ Nurse
- ◯ Physician
- ◯ Other (please specify)

[ ]

* 1.2 How many years of experience do you have in pre-hospital care?

* 1.3 What is your highest education qualification achieved to date?

- ◯ Doctoral degree (e.g., PhD, EdD, etc.)
- ◯ Master's degree or other post-graduate qualification (e.g., MA, MSc, MBA, etc.)
- ◯ Undergraduate degree (e.g., BSc,)
- ◯ Diploma (e.g., Post-secondary diploma, advanced diploma, etc.)
- ◯ Other (please specify)

* 1.4 In what country do you work?

* 1.5 In which type of area does your pre-hospital service primarily operate? (Please select all that apply.)

- ☐ Urban area (large city or metropolitan areas)
- ☐ Suburban area (areas surrounding cities, semi-urban)
- ☐ Rural area (countryside)
- ☐ Isolated or Natural (remote or wilderness areas)

* 1.6 How many mass casualty incidents have you responded to?

* 1.7 When was the last time you were involved in responding to a mass casualty incident?

- ◯ Less than 6 months ago
- ◯ Less than 1 year ago
- ◯ Less than 2 years ago
- ◯ Less than 3 years ago
- ◯ More than 3 years ago
- ◯ I don’t know

* 1.8 What types of mass casualty incidents have you responded to? (Select all that apply)

- ☐ Natural incidents (e.g., earthquakes, floods)
- ☐ Transport Accidents (e.g., car accidents, aircraft crashes, train derailment)
- ☐ Intentional explosion (e.g., terrorist attacks bombings)
- ☐ Accidental explosions (Industrial explosions)
- ☐ Fires
- ☐ Mass Shootings
- ☐ Mass Stabbings
- ☐ Stampede (e.g., crowd crushes at concerts, sports, or religious events)
- ☐ Structural Collapses (e.g., building or stage collapses)
- ☐ Other (please specify)

* 1.9 Please indicate the total number of casualties for the incident you responding to, including both injuries and fatalities. (If you have responded to more than one incident, choose the incident with the largest number of casualties.)

- ○ 1-5 victims
- ○ 6–10 victims
- ○ 11–30 victims
- ○ 31–50 victims
- ○ 51–200 victims
- ○ More than 200 victims
- ○ I don’t know

* 1.10 What was your primary role in responding to an incident?

- ○ Incident commander
- ○ Medical commander
- ○ Safety officer
- ○ Triage officer
- ○ Liaison officer
- ○ Treatment officer
- ○ Transport officer
- ○ Communications officer
- ○ Search and rescue personnel
- ○ Medical Care Provider (e.g., responsible for triage or initial treatment)
- ○ Other (please specify)

Factors impacting pre-hospital time in trauma-related mass casualty incidents section

* 2.1 Which of the following represents the longest interval of pre-hospital care you have encountered during your response to a mass casualty incident?

- ◯ Response Time (from receiving the call to arriving at the scene)
- ◯ On-Scene Time (time spent providing care at the incident site)
- ◯ Transport Time (from leaving the scene to arriving at a medical facility)

* 2.2 Please indicate your level of agreement with the following statement regarding your experiences in mass casualty incidents.

	Strongly disagree	Disagree	Neutral – neither agree or disagree	Agree	Strongly agree	N/A
Geographical challenges, such as remote incident locations, are a common cause of pre-hospital delays.	◯	◯	◯	◯	◯	◯
Weather conditions have a negligible impact on pre-hospital time delays.	◯	◯	◯	◯	◯	◯
Poor communication within the emergency response team often leads to increased pre-hospital time.	◯	◯	◯	◯	◯	◯
Dispatch centre's failure to provide accurate incident information led to critical delays in the response and on-scene coordination of pre-hospital care services.	◯	◯	◯	◯	◯	◯
The inadequacy of non-human resources, such as stretchers and medical equipment, have been a notable factor causing delays in providing care during mass casualty incidents.	◯	◯	◯	◯	◯	◯
Bystanders or relatives distracting me at the scene were a significant						

factor in causing delays, thereby hindering the timely delivery of care during mass casualty incidents.	○	○	○	○	○	○
Securing the scene for safety or tactical reasons was a significant factor in delaying pre-hospital time during mass casualty incidents.	○	○	○	○	○	○
Restrictions in the scope of practice for providers delay the administration of certain interventions in prehospital settings, consequently impacting the timeliness of care delivery.	○	○	○	○	○	○
Triage and extrication procedures in a congested incident scene often prolong the pre-hospital time for patients.	○	○	○	○	○	○
Delays in pre-hospital care are often due to logistical issues with transport resources.	○	○	○	○	○	○
Long distances from the incident scene to the hospital were identified as a main factor causing delays in transporting patients to receive definitive care.	○	○	○	○	○	○
The lack of experience and knowledge of unique injuries (e.g., paediatric emergencies, burns) typically encountered in mass casualty incidents can lead to significant delays in critical response times.	○	○	○	○	○	○

2.3 If you have encountered any other situations leading to delays in care that have not been previously mentioned, please describe them below.

* 2.4 Please indicate your level of agreement with the following statement regarding your experiences in mass casualty incidents.

	Strongly disagree	Disagree	Neutral – neither agree or disagree	Agree	Strongly agree	N/A
Bystander willingness to help with casualty loading or offer basic life support is associated with shorter scene times.	○	○	○	○	○	○
Prior regular training whether by tabletop exercises, leadership training, focused drills, or larger scale simulation are essential in improving the efficiency of pre-hospital response times in actual mass casualty incidents.	○	○	○	○	○	○
Trained emergency non-healthcare personnel, such as police officers and firefighters, on triaging and offering first aid have a significant impact on reducing pre-hospital time delays.	○	○	○	○	○	○
Advanced technologies such as drones and artificial intelligence have the potential to significantly reduce pre-hospital time.	○	○	○	○	○	○
Transforming triage into a dynamic, ongoing assessment that integrates aspects of immediate care can shorten pre-hospital time.	○	○	○	○	○	○
Incorporating pre-hospital Emergency						

Medicine as a subspecialty and involving physicians in advanced care significantly enhances patient outcomes by reducing pre-hospital times.	○	○	○	○	○	○
Effective coordination and the mobilisation of human resources, such as the rapid and spontaneous arrival of off-duty staff, significantly enhance the capacity for surge response during mass casualty incidents, positively impacting pre-hospital care times.	○	○	○	○	○	○
Efficient pre-hospital and hospital coordination and knowledge of traffic and geographical conditions are crucial for minimising transport times.	○	○	○	○	○	○
Responding to previous Mass Casualty Incidents has significantly improved my ability to respond effectively and save time in subsequent incidents.	○	○	○	○	○	○
The incorporation of Helicopter Emergency Medical Services (HEMS) in the response to Mass Casualty Incidents (MCIs) significantly enhances the efficiency of pre-hospital care, leading to a reduction in overall response times.	○	○	○	○	○	○

2.5 If you are aware of any other situations that have led to rapid delivery of care and have not been previously mentioned, please describe them below.

Pre-hospital response and triage section

* 3.1 Which triage system was used by your pre-hospital service during mass casualty incidents? (Please select from the dropdown menu.)

* 3.2. Considering the specific triage tool used in your service, do you believe it effectively optimises care and minimises delays during mass casualty incidents?

- Yes
- No

3.2.1 If Yes, please explain why?

3.2.2 If No, please explain Why?

* 3.3 Based on your experience in responding to mass casualty incidents, which life-saving interventions could be considered for early implementation during pre-hospital triage?

- [ ] Rapid Sequence Intubation
- [ ] Supraglottic Airway Device (e.g., I-gel)
- [ ] Oral Airway Insertion
- [ ] Surgical Cricothyroidotomy
- [ ] Oxygen Administration (e.g., non-Rebreather Mask)
- [ ] Finger Thoracostomy
- [ ] Needle Decompression
- [ ] Chest Tube
- [ ] Intravenous (IV) Access
- [ ] Oral analgesia (e.g., Oral Transmucosal Fentanyl Citrate)
- [ ] Intraosseous (IO) Access
- [ ] Tourniquet Application (e.g., CAT)
- [ ] Administration Of Tranexamic Acid (IM)
- [ ] Fluid/Blood Product Transfusions
- [ ] Haemostatic Agent (Celox Gauze)/Wound Packing
- [ ] Direct Pressure for Bleeding
- [ ] None - No interventions are recommended
- [ ] Other (please specify)

3.3.1 Based on the interventions you selected, please explain why you considered them essential for use in pre-hospital triage during the mass casualty incident(s) you responded to?

* 3.4 Based on your experience in responding to mass casualty incidents, which life-saving interventions are least likely to be implemented during pre-hospital triage?

- [ ] Rapid Sequence Intubation
- [ ] Supraglottic Airway Device (e.g., I-gel)
- [ ] Oral Airway Insertion
- [ ] Surgical Cricothyroidotomy
- [ ] Oxygen Administration (e.g., non-Rebreather Mask)
- [ ] Finger Thoracostomy
- [ ] Needle Decompression
- [ ] Chest Tube
- [ ] Intravenous (IV) Access
- [ ] Oral analgesia (e.g., Oral Transmucosal Fentanyl Citrate)
- [ ] Intraosseous (IO) Access
- [ ] Tourniquet Application (e.g., CAT)
- [ ] Administration Of Tranexamic Acid (IM)
- [ ] Fluid/Blood Product Transfusions
- [ ] Haemostatic Agent (Celox Gauze)/Wound Packing
- [ ] Direct Pressure for Bleeding
- [ ] None - No interventions are recommended
- [ ] Other (please specify)

3.4.1 Based on the interventions you selected, please explain why you think those life-saving interventions are least likely to be implemented during mass casualty incidents?
