# Supplementary material for: Pre-hospital care delays in trauma-related mass casualty incidents: a comparative international survey of high-income and low- and middle-income country perspectives
Source: Scand J Trauma Resusc Emerg Med. 2026 Jul 24;34:166. doi: 10.1186/s13049-026-01664-8 (PMC13692905; doi:10.1186/s13049-026-01664-8)
Supplement: Supplementary file 3 [file 13049_2026_1664_MOESM3_ESM.docx]

**Additional file 3:** Comparison of MCI types and casualty counts by income group.

^Panel (A) illustrates the distribution of MCI types based on participant responses, while Panel (B) presents the total number of casualties per event as reported by participants. Percentages represent the proportion of responses within each income group. An asterisk indicates statistical significance for p-values based on Fisher’s Exact Test or Chi-Square Test, with p < 0.05 (*), p < 0.01 (**), and p < 0.001 (***).^
