# Supplementary material for: Pre-hospital care delays in trauma-related mass casualty incidents: a comparative international survey of high-income and low- and middle-income country perspectives
Source: Scand J Trauma Resusc Emerg Med. 2026 Jul 24;34:166. doi: 10.1186/s13049-026-01664-8 (PMC13692905; doi:10.1186/s13049-026-01664-8)
Supplement: Supplementary file 4 [file 13049_2026_1664_MOESM4_ESM.docx]

**Additional file 4:** Comparison of the longest pre-hospital time intervals by income group.

^Abbreviations: HIC; High-Income Country, LMIC; Low- or Middle-Income Country.^

^Panel (A) represents the proportion of participants who identified Response Time as the longest interval, Panel (B) represents those who selected On-Scene Time, and Panel (C) represents those who chose Transport Time. Percentages reflect the proportion of responses within each income group. An asterisk indicates statistical significance for p-values based on Fisher’s Exact Test or Chi-Square Test, with p < 0.05 (*), p < 0.01 (**), and p < 0.001 (***).^
