# Supplementary material for: Pre-hospital care delays in trauma-related mass casualty incidents: a comparative international survey of high-income and low- and middle-income country perspectives
Source: Scand J Trauma Resusc Emerg Med. 2026 Jul 24;34:166. doi: 10.1186/s13049-026-01664-8 (PMC13692905; doi:10.1186/s13049-026-01664-8)
Supplement: Supplementary file 5 [file 13049_2026_1664_MOESM5_ESM.docx]

**Triage effectiveness**

Participants reported using more than 18 different MCI triage systems globally, with the START system being the most commonly used (46.43%), followed by the Standard Sieve (13.8%), while the remaining systems were used by 5% or fewer respondents. Overall, 72.8% of PHCPs expressed the opinion that the current triage systems were effective, with most systems having more respondents answering “yes” than “no,” except for the Standard Sieve and MPPT, where the “no” responses were higher.

Seven themes emerged regarding why PHCPs believe the triage systems are effective (Figure below). Of these, 39.5% highlighted the simplicity and ease of use of the system, noting that its effectiveness is enhanced by requiring minimal training and being accessible to a wide range of emergency teams. The second theme contributing to the effectiveness of triage systems is the prioritization of care and resource management 30.9%, ensuring that available resources are directed toward the most critically injured patients while reducing chaos and minimizing the time spent on those with less severe injuries. Additionally, 28.3% of PHCPs emphasized the familiarity and standardization of the systems, particularly in terms of their nationwide adoption and consistent use by all responders. Rapid performance of the triage tool emerged as a theme, with roughly 25% of PHCPs responding to the open-text question, indicating that the tool optimizes evaluation time and allows for quicker treatment of other urgent cases. The remaining themes, reported by less than 8% of respondents, included the use of a common language within the systems to facilitate communication with others. Another theme was adaptability and flexibility, highlighting the system’s ability to be applied in different scenarios. Additionally, there was some emphasis on how these tools help reduce cognitive load and stress.

On the other hand, seven additional themes were identified as reasons why the triage systems were perceived to be ineffective. The most frequently mentioned theme, cited by 28.9% of respondents in the open-text questions, was the complexity and lack of flexibility of the systems. Common phrases used to describe this theme emphasized the sophistication of some tools, with respondents calling them *“onerous to use”,* *“too complicated”,* *“too complex”,* or *“impossible to apply in an MCI”*. In terms of flexibility, feedback included phrases such as *“trauma-centric and a very blunt tool and needs to incorporate dynamic assessment, not remain fixed”.* Secondly, the reliance on clinical judgment and experience was identified as another key theme 26.3%, with respondents expressing concern that these tools default to anatomical triage, potentially preventing PHCPs from relying on their “gut feelings” for clinical assessment. This reliance on rigid systems highlights the lack of clinically nuanced decision-support tools. Following that, three themes, each reported by 15.8% of respondents, highlighted inefficiency and slow response, lack of adaptability to different types of incidents, and limitations related to incident size and event scale. Regarding slow response, common phrases centered around the tools being “too slow to carry out as a solo responder” or “largely abandoned and not used.” The lack of adaptability to different types of MCIs was noted, with respondents emphasizing that these tools are focused primarily on trauma, leaving other incidents such as CBRN (Chemical, Biological, Radiological, and Nuclear) events uncovered. For limitations related to incident size and event scale, some respondents pointed out the tools’ inefficacy in large-scale incidents, while others noted challenges in smaller-scale events, suggesting there is a need for more nuance in patient categorization. The remaining minor themes included challenges with categorization and prioritization (13.2%), such as issues with over- or under-triage, and insufficient training and practice (10.52%). One respondent described the latter by stating, *“It’s always like the first day of school when using it. Everyone is trying to figure out where to go when the bell rings”*.

**Additional file 5:** Thematic representation of the respondents’ perceptions of the effectiveness of triage tools used during MCIs.
