# Supplementary material for: Pre-hospital care delays in trauma-related mass casualty incidents: a comparative international survey of high-income and low- and middle-income country perspectives
Source: Scand J Trauma Resusc Emerg Med. 2026 Jul 24;34:166. doi: 10.1186/s13049-026-01664-8 (PMC13692905; doi:10.1186/s13049-026-01664-8)
Supplement: Supplementary file 6 [file 13049_2026_1664_MOESM6_ESM.docx]

**Pre-hospital life-saving interventions used during MCI triage response**

Participant feedback regarding interventions revealed that basic interventions were more likely to be implemented than advanced interventions during MCI triage (Figure below).

Among the more likely interventions, tourniquet application (66.66%) and direct pressure for bleeding (59.56%) showed the largest practical differences but did not achieve statistical significance (*p*= 1.00 and *p*= 0.162, respectively). Conversely, interventions such as hemostatic agents (38.67%), oxygen administration (29.78%), and needle decompression (32.00%) were statistically significant (*p* < 0.05).

Among the less likely interventions, advanced interventions such as surgical cricothyroidotomy (43.11%) and chest tube insertion (40.00%) displayed large practical differences; however, surgical cricothyroidotomy was not statistically significant (p = 0.1585), whereas chest tube insertion was statistically significant (p = 0.0077). However, rapid sequence intubation (33.77%) and finger thoracostomy (20.89%) were statistically significant (*p* < 0.05), while fluid/blood product transfusions (11.55%) were less likely to be implemented despite strong statistical significance (*p* < 0.001).

 **Additional file 6:** Likelihood of implementing life-saving interventions during pre-hospital MCI triage.

^This bar chart shows the percentage of respondents (out of 225) who considered specific life-saving interventions as either essential (more likely) or least likely to be implemented during pre-hospital triage in MCIs.^

^*Black asterisks indicate interventions significantly more likely to be implemented, and red asterisks indicate those significantly less likely to be implemented. Statistical significance was determined using Chi-Square or Fisher’s Exact Test (p < 0.05).^
